# Promoting Physical Activity to Cancer Survivors in Practice: Challenges and Solutions for Implementation

**DOI:** 10.3390/cancers17050850

**Published:** 2025-02-28

**Authors:** Sarah Hardcastle, Patricia Sheehan, Bróna Kehoe, Michael Harrison, Mairéad Cantwell, Niall Moyna

**Affiliations:** 1 School of Sport and Physical Activity, Sheffield Hallam University, Sheffield S10 2BP, UK; 2 Institute for Health Research, University of Notre Dame Australia, Fremantle, WA 6160, Australia; 3 Department of Sport and Exercise Science, South East Technological University, X91 HE36 Waterford, Ireland; patricia.sheehan@setu.ie (P.S.); brona.kehoe@setu.ie (B.K.); michael.harrison@setu.ie (M.H.); 4 Department of Sport & Health Sciences, Technological University of the Shannon, N37 HD68 Athlone, Ireland; mairead.cantwell@tus.ie; 5 School of Health & Human Performance, Dublin City University, D09 V209 Dublin, Ireland; nial.moyna@dcu.ie

**Keywords:** adherence, behaviour change, cancer survivors, exercise, oncology

## Abstract

**Objectives**: This study explored the challenges and solutions to the effective promotion of physical activity (PA) to cancer survivors in practice in Ireland amongst a variety of stakeholders, including healthcare professionals (HCPs), policy makers, community providers of cancer care, and academics. **Methods**: Focus groups (n = 5) were conducted with participants (n = 40), including oncologists, oncology nurses, physiotherapists, cancer support centre managers, academics, consumers, cancer policy representatives, exercise specialists, community providers of exercise programmes, and a chartered psychologist. The data were analysed using reflexive thematic analysis. **Results**: Four primary themes were identified: (i) embedding PA into the cancer pathway (including the sub-themes of ‘singing from the same hymn sheet’, ‘PA as an essential element of treatment’, and ‘intervention opportunities and models of care’); (ii) education and training; (iii) access to appropriate PA interventions; and (iv) tailored programmes. **Conclusions**: The importance of embedding PA into the cancer pathway such that PA is viewed by clinicians and patients as a key part of their treatment was emphasised. Opportunities for PA intervention within existing practice were highlighted, such as the chemotherapy education sessions run by Daffodil nurses. Findings pointed to a need for education and training for HCPs that include how to coach and counsel cancer patients concerning PA in clinic. Finally, a major barrier to PA promotion was limited access to appropriate PA interventions.

## Simple Summary

Despite evidence of the importance of physical activity (PA) for healthy cancer survivorship, PA is not systematically promoted in practice. The present study sought to identify the challenges and solutions concerning the effective promotion of PA to cancer survivors in routine practice amongst a broad range of stakeholders in Ireland. Focus groups were conducted with participants (n = 40), including oncologists, oncology nurses, physiotherapists, cancer support centre managers, academics, consumers, policy representatives, and exercise specialists. The findings emphasised a need for HCP training, including PA counselling and the development of a PA care pathway. The main barrier to PA promotion was limited access to PA programmes. The dominant model was facility-based exercise programmes with considerably less focus on home-based PA. Worthwhile intervention efforts included the promotion of lifestyle-based PA and offering a diversity of PA interventions that align with the varying exercise preferences and support needs of cancer survivors.

## 1. Introduction

There is substantial evidence for the role of physical activity (PA) for healthy cancer survivorship trajectory across many cancers [1,2,3]. Being physically active post diagnosis and post treatment is vital to reduce the risks of cancer-specific and all-cause mortality and improve survival [2,4]. Survivors who are physically active have lower CVD-related morbidity [5], lower recurrence risk [2], and improved survival compared to those who are inactive [2].

Given the benefits of PA in cancer, several international guidelines recommend PA for those living with and beyond cancer [6,7,8]. The guidelines recommend that adult cancer survivors participate in 150 min of aerobic moderate-intensity PA per week (or 75 min per week of vigorous-intensity PA) and muscle strengthening activities on two or more days a week [6]. However, few cancer survivors achieve these guidelines [9]. Therefore, it has been recommended that PA be promoted by oncologists and HCPs as part of routine care [10] (For the purposes of this study, PA and exercise are used interchangeably. However, the focus is on PA that is consistent with the international guidelines for cancer survivors (i.e., aerobic PA that is at least moderate intensity)). This is due to their ability to reach all patients and promote PA and because patients place trust and confidence in their healthcare providers, particularly their treating oncologists [10,11,12]. Further evidence suggests that cancer survivors who recall receiving PA advice from their oncologist are more likely to achieve the PA guidelines [13,14]. Despite this, few oncologists and HCPs routinely promote PA to cancer survivors in practice [15,16].

There is a relative dearth of research concerning the challenges and potential solutions to PA promotion amongst HCPs and allied professionals. Previous research has identified the following barriers to routine PA promotion: a lack of time [15,16,17]; a lack of exercise programmes [17,18]; a lack of educational materials [15,17,18]; insufficient specific exercise knowledge [16,17,19,20] or confidence in PA counselling [21,22]; a lack of access to exercise professionals [19,23]; and perceived lack of interest in or motivation for PA amongst patients [15,17,24]. The facilitators of PA promotion have been less well explored [25] and include access to expert exercise professionals [16,19,21,26]; patient educational materials [16,19,26]; electronic referral forms [16,19,26]; and education on the PA guidelines and training for HCPs in PA promotion [21,27]. However, the existing knowledge to date has been derived primarily from surveys [25] that may not capture the complexity of the matter. Further, most surveys have tended to use predetermined categories to examine barriers to PA promotion, thereby limiting new findings concerning challenges and solutions. Further inductive work is needed to explore the challenges to PA promotion in cancer care and potential solutions to help support PA promotion in practice. To our knowledge, this is the first study that focuses on challenges and solutions to PA promotion from a broad range of stakeholders involved in cancer care across hospital and community settings including oncologists, nurses, physiotherapists, exercise professionals, cancer survivors (consumers), policy makers, cancer support centre managers, community exercise providers, and academics. The aim of the present study was to identify the challenges and solutions concerning the effective promotion of PA to cancer survivors in routine practice amongst a broad range of stakeholders.

## 2. Materials and Methods

### 2.1. Participants

Participants for the study were recruited from a previous stage of a larger project reviewing the PA and exercise initiatives available for cancer survivors within the Republic of Ireland (following a successful tender awarded by the National Cancer Control Programme (NCCP) in 2022). The earlier stage of the project involved scoping out PA programmes and services and involved setting up meetings with cancer support centres (CSCs) (n = 46 nationally), known community exercise providers, academics working within exercise oncology in Ireland, and oncology professionals working within hospitals (primarily nurses and physiotherapists). Participants from the earlier stage of the project that had consented to be contacted again to participate in a roundtable discussion were invited to participate. The only additions were oncologists that were sought out to participate in the discussion and consumer representatives (i.e., cancer survivors). Oncologists were invited on the basis that they were known to have an interest in exercise oncology and practiced in Ireland. Consumer representative contact details were provided by the NCCP through their PA and exercise subgroup and had consented to participation in the discussions prior to their contact details being shared by the NCCP with the research team.

### 2.2. Procedure

The current study conformed to the Standards for Reporting Qualitative Research [28] (see online Supplementary File S1). Dublin City University Research Ethics Committee approved this study (#2022/093). The first author contacted participants who expressed a willingness to participate, and an interview date was arranged. Participants received an information sheet, provided informed consent prior to the interview, and were informed that their identity would be concealed in any reporting of the data.

Five focus group discussions were conducted between July and September 2022. Forty unique participants were recruited and participated in a single discussion. Each roundtable contained between 7 and 10 participants, with a balance of the different stakeholder roles represented at each (e.g., oncology nurse, consumer representative, NCCP representative, physiotherapist, cancer support centre manager, exercise specialist, academic). The range of stakeholder engagement was achieved through careful planning and trying to ensure one of each type of stakeholder would be represented at each discussion. Doodle polls were used to ascertain availability prior to confirming a date for each discussion. A target sample size of 40, covered in 5 roundtable discussions, was considered an adequate number to obtain various perspectives on the ways forward regarding the expansion of exercise programmes and PA services in Ireland for cancer survivors. We did not set out to reach data saturation per se, since the concept of saturation is contested and founded on neo-positivist leanings in qualitative research [29]. According to Low [30], the notion of data saturation is a fallacy, since there are always new insights to be gained from further data collection and analysis. Instead, the focus was on obtaining multiple perspectives across differing stakeholders as a starting point to understanding the main challenges and ways forward in this area. In this way, sample size was based more on the concept of information power [31]; that is, the more relevant information a sample holds, the fewer participants are needed. Nevertheless, it is recognised that when no new information is derived from further data collection, this may also be a further indicator of data sufficiency. Indeed, previous research observed that most themes (90%) identified were generated within between 3 to 6 focus groups, and that 3 focus groups were sufficient to identify all the most prevalent themes within the dataset [32].

These were conducted via Zoom and audio-recorded (with permission) using an Olympus digital voice recorder (WS-811). Each discussion lasted for approximately two-hours and was led by the first author who has a wealth of experience in qualitative data collection and analysis and is an active researcher in exercise oncology. Participants did not know the focus group facilitator except for crossing paths in a professional capacity in an earlier stage of the project. Participants were aware that the facilitator was an academic working with a research team scoping out national provision of PA programmes and services for cancer survivors, and that this project was funded by the NCCP. Each discussion began with brief introductions followed by the first author providing a summary of the findings from the scoping review (i.e., existing provision of PA programmes and services in Ireland for cancer survivors) and providing clarity on the target behaviour (i.e., the promotion of PA that is at least moderate-intensity for at least 150 min per week, consistent with the guidelines) before opening for discussion on the ways forward to promote PA more effectively to cancer survivors in practice. The discussion on the ways forward in this area was primarily inductive; although, Table 1 provides some examples of the questions posed during the discussions. The facilitator ensured that all voices were heard and actively invited comments from participants that did not volunteer experiences, challenges, or solutions. It is recognised that the facilitator is an active researcher within exercise oncology and has published specific ideas concerning PA promotion to cancer survivors [10] that may have influenced data analysis and interpretations. However, another member of the research team (NM) acted as a joint facilitator in focus group discussions to ensure multiple perspectives were gleaned and to help prevent the over-steering of the discussion by the primary facilitator. Further, the systematic coding of transcripts helped to ensure that themes were grounded in the data. Interviews were digitally recorded and transcribed verbatim using a professional transcription service. The study adopted a social constructionist reflexive thematic analysis [33], underpinned by a realist ontology. An ontological realist position assumes that the data can inform us of something about reality; although, acknowledging not absolutely mirroring it, as it is filtered through the lens of experience and meaning including the analysis process by the researcher [34]. The epistemological position adopted was one social constructionism (i.e., recognising that patterns identified are socially constructed) with the researcher active in the creation of meaning and themes.

### 2.3. Data Analysis

The data were analysed by the first author using reflexive thematic analysis [33] to generate themes. Analysis included deductive and inductive approaches; whilst a codebook was not adopted, and categories were not pre-determined, it is recognised that the interview guide focused primarily on the challenges and solutions to promoting PA, and therefore, analysis was not entirely inductive. Nevertheless, the data were ‘open-coded’ to best represent the perceptions and experiences as conveyed by participants [35]. Thematic reflexive analysis involved several steps, including (i) immersion and the careful reading of transcripts; (ii) attaching codes to salient text segments; and (iii) the identification of themes at a broader level and examining whether codes may be combined to form an overarching theme. During these processes, inductive analysis was used to generate themes grounded in the data. The final step involved reviewing themes, cross-checking for overlap, and finally, defining and classifying themes. Codes were generated from each focus group discussion separately prior to conducting a cross-focus group analysis. Supplementary File S2 provides an overview of the list of codes from each roundtable discussion and a list of codes generated through the cross-focus group analysis. The analysis offered is one interpretation of the data, and other interpretations are possible. Nevertheless, we aim to offer a credible and trustworthy interpretation that accurately captures the data. For example, we provide a ‘thick description’ via the use of extensive quotations, so that the reader can evaluate the interpretation.

**Table 1:** Roundtable discussion guide.

Discussion Guide	
	How can physical activity (PA) services for cancer be expanded in Ireland? What are the main challenges to promoting PA to cancer survivors? How could gaps between research and practice could be bridged? (Facilitator gives some examples of research-based programmes and existing provision in the community.) How could a more systematic approach to exercise promotion be set up? What would it look like? How could we create more direct links between hospital and community-based exercise programmes? How could referral be optimised? Who should/could refer? How could we more effectively implement exercise programmes and PA services for cancer patients? Including recommendations for different points on the patient pathway (i.e., prior to surgery or treatment, during treatment, following treatment) How could we create more evidence-based PA promotion? What about adherence? Could we ask survivors about their experiences in relation to PA promotion during their cancer journey? How to deal with conflicting findings? e.g., both Cantwell et al. [17] and Hardcastle et al. [15] found that clinicians cite low motivation or interest from cancer survivors concerning PA. However, several studies with cancer survivors (e.g., Maxwell-Smith et al. [36]; Hardcastle et al. [12] found consistently that patients want to receive PA information and advice from their oncologist. How other clinician barriers to PA promotion could be overcome (e.g., limited time with patients; lack of community-based exercise rehabilitation programmes to refer to; lack of resources regarding physical activity for cancer survivors (e.g., education leaflets and materials; lack of knowledge regarding physical activity prescription for cancer survivors; patients’ family/friends advise patients to rest and avoid activity).

## 3. Results

**Figure 1:**
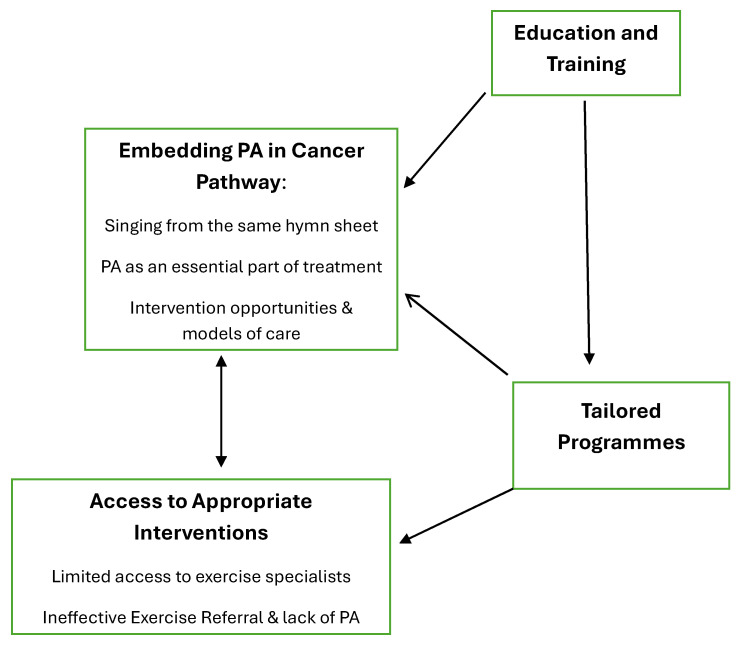
Thematic map of relationship between themes.

In total, 40 participated in roundtable discussions. Table 2 provides an overview of participant characteristics. Data analysis generated four primary themes and five sub-themes: (i) embedding PA into the cancer pathway (sub-themes: ‘singing from the same hymn sheet’, ‘PA as an essential element of treatment’, and ‘intervention opportunities and models of care’); (ii) education and training; (iii) access to appropriate PA interventions (sub-themes: ‘limited access to exercise specialists’ and ‘ineffective exercise referral and lack of PA services’; and (iv) tailored programmes. Table 3 provides an overview of themes and additional quotes, and Figure 1 provides a thematic map to illustrate the relationship between themes. The importance of embedding PA into the cancer pathway was the overarching theme, whereby participants stated that policy changes would be needed to ensure that (i) HCPs are consistent in PA messaging to patients, (ii) PA becomes integrated into cancer care to become usual care, and (iii) a PA model of care is developed and implemented. The second theme, ‘education and training’, is closely related to the overarching theme, since it was established that many involved in cancer care do not know the PA guidelines, the value of PA for cancer survivors, or how to motivate or engage patients in relation to PA. The third theme ‘access to appropriate interventions’ concerns the lack of PA programmes and services available for cancer survivors, including difficulties accessing PA specialists and poor uptake to and attrition of services that do exist. The theme on the need for appropriate intervention is closely linked to the final theme on the need for tailored interventions, according to patient exercise preferences and support needs.

### 3.1. Embedding PA into the Cancer Pathway

The dominant theme was the importance of embedding PA into the cancer pathway and the importance of oncologists in PA promotion: “This all has to emanate from the consultant. If it’s not coming from top down, I feel there’s no buy in” (academic 1) and “It’s that top down…from the oncologists to engage and the nurse specialists who are dealing quite intimately with the patients…that’s where the core of information will come” (physiotherapist, hospital 15). Participants stated unequivocally that PA should be embedded as a key part of cancer treatment and assessed in the same way that other clinical parameters are measured as standard care: “It should become embedded as a key element of care, just like measuring your blood pressure or checking your blood count” (community provider of exercise programmes (CPEP) 1). However, PA may not be a high priority to oncologists: “The doctors have different views…some people view this as not a priority” (medical oncologist, hospital 1). Participants believed that the NCCP played an essential role in reaching oncologists: “It has to be through the NCCP…if we have the buy in through the NCCP we will get the oncologists” (cancer nurse specialist (CNS), hospital 1). The theme of ‘embedding PA into the cancer pathway’ contained three distinct sub-themes that represented key concepts that underpinned the overall theme; ‘singing from the same hymn sheet’ represented the importance of consistent messaging on PA from all those involved in delivering cancer care; ‘PA as an essential element of treatment’ represented the view that PA should be part of usual care (assessed, promoted, prescribed) and a form of treatment (i.e., exercise as medicine), alongside other standard treatments, such as chemotherapy or radiotherapy. The final sub-theme ‘intervention opportunities and models of care’ is related to how PA could be embedded into the cancer pathway. Therefore, the three elements of the theme are related in that PA promotion in cancer care is likely to need consistent messaging, be viewed as an essential element of usual care (i.e., integrated into the cancer pathway), and will need embedding into the cancer pathway through the development of a care pathway and identifying opportunities for intervention.

#### 3.1.1. Singing from the Same Hymn Sheet

It was deemed important that all HCPs were involved in the promotion of PA: “We need to get all of the oncologists and oncology nurses on board…it’s not just oncologists, it’s the surgeons…the radiation oncologists, all of these people have a role to play in promoting exercise” (advanced nurse practitioner (ANP), hospital 1), and “We have to have clinicians stressing the importance of this whether it is the consultant, the nurse or anybody in the multi-disciplinary team (MDT)…The MDT stressing the importance of exercise” (NCCP 1). It was also seen as important that HCPs and allied support workers all sing from the same hymn sheet: “It requires absolutely everybody from day one to be saying the same thing” (ANP, hospital 1). The continuity in PA messaging was viewed as particularly important given a consumer’s recollection of negative feedback on her exercise participation by a CNS “It’s more negative, you should be taking it easy” (breast cancer survivor 1). The importance of consistent messaging to patients by the whole oncology team was underlined: “You can’t underestimate the value of your nursing staff…when someone is getting chemotherapy, they’re in the day ward for a number of hours…they’re talking to the nurses… [the nurses could] reiterate the message that it is ok to exercise and giving that encouragement” (academic 4).

#### 3.1.2. PA as an Essential Element of Treatment

All participants believed that PA should be an essential part of cancer treatment that is integrated into the cancer care pathway: “To evoke/embed exercise as an essential part of the pathway” (CPEP 2), and “An ANP makes it almost mandatory for the patient to say ‘you are finished with your treatment part and now I want you to go to this end of treatment workshop so that is your next appointment” (NCCP 2). In further reference to PA being essential rather than an adjunct treatment, several participants stated that PA should be prescribed to patients with an appropriate rationale provided, whether this be the role of PA to alleviate treatment-related side effects or the role of PA in the survivorship phase in terms of reducing cardiovascular disease risk or risk of cancer recurrence. For example, “We’re prescribing this as a measure to counteract and limit some of the side effects of treatment initially” (physiotherapist, hospital 10), and “This is what you’re supposed to be doing…exercise is safe and not only is it safe but you have to exercise…if you don’t exercise you know you’re more at risk of recurrence” (physiotherapy manager, hospital 7). One oncology unit provides exercise prescriptions to patients: “We print it out and give it when we are discussing chemotherapy or hormone therapy” (medical oncologist, hospital 1). Prescribed exercise was also advocated by a consumer: “My initial meeting with my oncologist and my radiation oncologist both said…the best things you can do for yourself are keeping active and keeping your weight down…but I heard them at the beginning and never again…It really needs to be prescribed” (breast cancer survivor 2).

#### 3.1.3. Intervention Opportunities and Models of Care

Potential intervention opportunities and other models of care that could be translated to the PA domain was a sub-theme of embedding PA into the cancer pathway. One such intervention opportunity is the integration of PA promotion within the chemotherapy education session: “We do chemo education, and I would spend usually up to an hour with the patient…I give many a week, I have never been asked about exercise. We cover it. We normally tell people to keep moving, keep to the level you are at if you can, but it is a sentence or two in an hour, there is something to be done there I think” (CNS, hospital 3). The potential of such sessions was echoed by a NCCP representative: “The daffodil nurses in all centres do a chemo education session at the start of treatment…maybe we could be stronger in our messaging as regards to exercise… even using some of the pieces you have talked about earlier in terms of time [minutes] and you know and importance of the type of moving [exercise intensity]” (NCCP 2). The meeting with the physiotherapist following surgery was also identified as an intervention opportunity to promote PA behaviour change rather than short-term mobility: “When I was in for surgeries you’d have a visit from physio and they go through the exercise…to stretch and that…there would be a real opportunity there for a physio to give people permission to start walking…so it’s not just your exercise for the next 10 days or whatever” (breast cancer survivor 1).

Others referred to existing models in cancer care including that for psycho-oncology, which adopts a stepped care approach to intervention. It was noted that existing programmes (primarily supervised) were not required for all survivors: “Everybody doesn’t need a structured programme and I would be going towards a model of care (like) psycho-oncology…that is stepped so we know we’re going to start off for people who just need written information, advice or recommendations from HCPs” (physiotherapist, hospital 8). A stepped and innovative care approach was also highlighted as crucial to reach the large number (i.e., 44,000) of newly diagnosed cancer patients every year: “We don’t have anywhere near the capacity to take 44,000 patients…We have to [be] pragmatic and take a staged approach here. It is obvious that centre-based facilities probably just won’t work…so we have to be innovative in how we can facilitate PA” (academic 1).

### 3.2. Education and Training

This theme concerned the need for training for HCPs and allied workers concerning the importance of PA for cancer survivors, knowledge of the guidelines, and appropriate PA primarily. It seems that patients do not tend to receive written information or specific recommendations concerning PA: “There’s very much a lack of information regarding exercise…no oncologist or breast surgeon or nurse suggested it to me” (breast cancer survivor 2).

Cancer survivors may also not be aware of the importance of PA for their health or survivorship trajectory: “A lot of patients don’t realise the importance of exercise when they are diagnosed” (manager, CSC 1). Related to education on the PA guidelines, there were concerns raised regarding some of the physical activities offered at CSCs that do not align well with the PA guidelines: “At our local centre they have yoga or Pilates but that is not meeting your activity guidelines…you can’t put them in the same bracket as getting your cardiovascular exercise and the importance of that imparted to the patient” (physiotherapy manager, hospital 7). The first author shared with participants that yoga was the most common PA provided at CSCs, offered at 14 CSCs (out of 20 CSCs that offered PA opportunities or programmes). Participants believed this reflected the perception that survivors should only participate in gentle exercise: “I think that highlights that we still tend to think that people are not able to do that 150 min [of moderate-to-vigorous PA]…Yoga is provided because it’s seen as gentle sport…a kind of gentle way of half exercising, but not going to hurt them…I think that sends the wrong message” (CNS, hospital 3). Another nurse explains that clinicians, nurses, and CSC staff would benefit from receiving further education and training: “I am only trying to learn all of this myself by my own interest in it. Whereas I suppose if doctors and nurses and the CSCs and all those kinds of supports have had some sort of a module that they could attend to learn all of this, it would be really useful to promote the awareness piece…HCPs actually need some form of module or CPD around exercise guidelines and the programmes available and how to coach and mentor your patient in a clinic in relation to exercise” (ANP, hospital 1). Another highlighted the need for further training on how to motivate patients for PA behaviour change that are not expert driven: “Training HCPs such as nurses in how to facilitate and give these programmes…snippets of techniques like motivational interviewing, teaching those types of coaching approaches to our medics and nursing staff and physio staff rather than the expert approach ‘you should exercise’” (CNS, Hosp 1).

### 3.3. Access to Appropriate PA Interventions

Access to appropriate PA interventions included two sub-themes: (i) limited access to exercise specialists and (ii) ineffective exercise referral and lack of PA services.

#### 3.3.1. Limited Access to Exercise Specialists

Limited access to appropriate personnel to lead PA programmes was considered a barrier to provision. This access issue was raised primarily by those managing CSCs: “Access to proper professionals…we’re not experts in exercise…we have to outsource exercise and there’s no drive from gyms to run courses” (manager, CSC 3), and “The main challenges are the funding to run programmes and getting physiotherapists can be difficult” (manager, CSC 10). Another refers to the unequal geographical distribution of exercise specialists in Ireland: “We serve seven counties, but people may be living in Donegal and there may not be a service where an exercise specialist who specialises in cancer is there to support those people” (exercise specialist, CSC 2). Limited access to exercise specialists was also related to the cost incurred in the provision of PA programmes: “The only thing that is stopping us is finances…getting the physio in more often. She’s here two days a week… [and the] financial difficulty of offering exercise programmes: ExWell charge” (manager CSC 6) (ExWell Medical is the primary national provider of community-based exercise programmes. They offer several programmes. Two CSCs refer patients to a supervised twice weekly, 12-week supervised programme (not cancer specific) at a cost of €220. ExWell Medical also provide a ‘Move on’ supervised exercise programme for cancer survivors at a cost €70 per month for 8 classes or €10 per class).

#### 3.3.2. Ineffective Exercise Referral and Lack of PA Services

This sub-theme included low rates of referral to community exercise programmes, such as those offered by ExWell and those offered through CSCs, and the need for a simple referral mechanism and a clear referral pathway. Participants noted generally low rates of referral for exercise: “Oncologists sometimes refer for general support but not exercise directly” (manager, CSC 11), and “Getting referrals from oncologists or GPs. PA is not on their agenda. It is not highly valued…we need to find a way to sell exercise to clinicians” (manager, CSC 2). Low rates of referral were also noted by the NCCP: “Across a number of initiatives referrals can be quite low from the hospital settings to programmes in the community…so we are looking at other options like self-referral being allowed to programmes” (NCCP 3). Self-referral protocols may be problematic, because they are likely to attract sufficiently physically active survivors, rather than targeting those who are insufficiently physically active. For example, “Our referrals are where people have an interest in exercise…so we refer to ExWell where somebody during our introduction conversation expresses an interest in exercise…we are not prescribing it” (manager, CSC 13).

A simple and quick referral mechanism was noted as an important step to improve exercise referral rates: “It has to be made as simple as possible—that there is no cumbersome paperwork, or licking stamps or putting them on letters, you know, it’s like Health Link… just press a button and it is done” (CPEP 2). An opt-out exercise referral was also viewed as useful: “Making the referral easy and also making the referral almost an opt-out rather than opt-in, that is an important concept” (CPEP 2). However, the opt-out option may not be effective if it bypasses the oncologist stating the importance of PA and explicitly stating that the patient is being referred: “We were quite shocked that we would phone somebody and they had never heard of us, even though they had been referred” (CPEP 2).

The establishment of a clear referral pathway was considered an important endeavour but so too was having the appropriate PA services in place to refer to: “If there’s not a referral pathway there from the consultants, they [the exercise programmes] won’t be as used as they need to be…we’re trying to build those links with the consultants here in Galway…It’s really a two-pronged approach…We need the referrals, and they need to have the services available too” (exercise specialist, CSC 2). It was highlighted that there were few exercise programmes to refer survivors to: “I really struggle with where to send them…if I had a magic wand it would be that those levels of support are there and then we keep those people who need it the most” (physiotherapist, hospital 10), and “You can get swamped quite quickly in potentially inappropriate referrals…not everyone with cancer might need a specialised group to come in to” (physiotherapist, hospital 15). There was a recognition that many survivors did not need a supervised hospital-based exercise programme, but that there was a gap in provision of appropriate PA programmes in the community setting: “I think a proportion of those (I’ve seen) should have been an assess and advice session and straight out into the community but when I struggle to link people with the community, then I have to keep them for a little bit longer” (physiotherapist, hospital 10).

### 3.4. Tailored Programmes

The final theme concerned the importance of tailored programmes that align with survivors’ exercise preferences and support needs. Tailored programmes included many aspects including the types of PA programmes offered to survivors with a recognition that “There is no one size fits all and I think we have to have the breadth, the flexibility that allows us to ensure that every individual, regardless of the circumstance, is going to get access to some form of intervention” (academic 1) and catering for varying exercise preferences: “To tailor it according to the needs of those patients…sometimes people like it in a group. Some people like it single…some online… some like it face-to-face. Some don’t want to sit in with a load of cancer survivors because they don’t identify themselves as cancer survivors. They just want to put it behind them and never think about it again” (medical oncologist, hospital 12). Some will not want to participate in exercise programmes alongside other cancer survivors: “I was told that very forcibly by a metastatic cancer patient…that the last thing she wants is to walk into a room and see other frail, bald, wearing scarves patients doing exercise who have cancer” (CPEP 2).

The dominant model has been the delivery of facility and group-based exercise programmes for cancer survivors. However, poor uptake and high attrition can be a problem in such group-based programmes: “Of those who attended, those who started and those who are currently active you’re down to 25% of the whole lot very quickly” (CPEP 2). Several reasons were provided for the low uptake and high attrition in such exercise programmes including cost, access, and exercise preferences. For example, cost was viewed as a barrier to participation: “Some wouldn’t have the budget to go to a gym” (prostate cancer survivor 3), and “Funding is a huge issue for my patients. I’d love them all to go to ExWell” (physiotherapist, hospital 10). Along with cost, logistical issues including access and availability of programmes were also noted as barriers to participation: “So many go back to work, and that affects our classes because they are not on in the evenings” (CPEP 3), and “A number of people on treatment are not able to drive…so that sort of disenfranchises them from being to attend gym sessions” (physiotherapist, hospital 1). It was also recognised that many survivors prefer individual rather than group-based programmes: “A lot of people work with me one-on-one. They don’t want groups” (physiotherapist, hospital 10).

## 4. Discussion

To our knowledge, this is one of the first qualitative studies to explore the challenges and solutions to promoting PA to cancer survivors nationally, across a broad range of stakeholders, including those working in clinical, community, and research settings, in addition to consumers and those involved in setting cancer policy. Our findings echo existing knowledge in calls to action to embed PA promotion into routine cancer care [3] from the point of diagnosis onwards with the NCCP playing an essential role in reaching the oncologists and ensuring that PA is integrated into the cancer pathway. There are four main contributions to knowledge generated from findings in the present study. Firstly, the finding related to consistent and repetitive PA messaging from all HCPs indicates the need for a whole systems approach to PA in cancer care. This is a contribution, since PA champions have been proposed to facilitate PA promotion in cancer care [19,37,38]. However, the impact of PA champions in healthcare settings may be somewhat limited without a whole systems approach, where a consistent message about the importance of PA is provided by the surgical or medical oncologist and continued by all who regularly encounter cancer survivors. Structural and policy change is needed to ensure that PA promotion is implemented in practice. The NCCP should ensure that guidance and recommendation for PA is standard of care for cancer patients through the development and implementation of a PA cancer care pathway. Such PA messaging needs to be clear and advocating the PA guidelines, rather than a vague message, such as ‘keep active’, as recalled in the present study. This is important, since cancer survivors may hold erroneous views about being sufficiently physically active [12,36] and place trust and confidence in the advice from HCPs, particularly their treating oncologists [10,11].

Secondly, the present study extends understanding in relation to education and training needs for HCPs and those involved in cancer care, finding that, in addition to a lack of knowledge of the PA guidelines being a barrier to PA promotion [16,17,19,20], HCPs also need training in PA counselling for use during routine consultations. Such PA counselling would include how to raise the subject of PA and motivate patients to engage in PA in a patient-centred manner in addition to education concerning the importance of PA for cancer survivors and its specific role at the various stages of the cancer journey (i.e., becoming fitter for surgery, alleviating or coping with side effects of treatment, and longer-term health). The contribution to and need for education also extends to the charitable sector with the finding that the most common PA offered to cancer survivors at CSCs was yoga, which, although popular, is unlikely in most cases to help patients to meet the PA guidelines.

The third contribution relates to understandings concerning exercise referral in cancer care. Consistent with previous research, participants emphasised a lack of PA programmes to refer or signpost to, and this has been identified previously [17,18,19,25]. However, the present study also found a lack of referral to existing exercise programmes. For example, it is notable that, where a well-established and medically based exercise programme for long-term conditions including cancer does exist in Ireland (i.e., that offered by ExWell Medical), there were relatively few referrals to it from HCPs. Findings from the present study suggest that the opt-out referral system proposed may not be effective without the robust messaging by clinicians, reinforcing the importance of PA and making the referral clear to patients during a consultation. Therefore, the present study extends previous findings, indicating that work needs to focus on increasing active referral (in addition to finding ways to simplify referral, i.e., with the use of electronic referral forms [16,19,26] with HCPs verbally emphasising the importance of PA to patients alongside referrals.

The fourth contribution of the present study is the unveiling of dominant thinking regarding PA promotion in cancer care that may be inadvertently limiting a diversification of approaches to PA promotion in practice. In the present study, the dominant model and mindset when considering PA provision was facility-based or comprised supervised exercise programmes, with considerably less focus on promoting self-management of PA or home-based PA. This is an important finding, given that findings from the present study, alongside previous research, identified that exercise preferences of cancer survivors vary, and many do not enjoy group-based programmes [39,40,41]. The cost, distance, and timing of exercise programmes were also noted as barriers to facility-based exercise programmes. Remotely delivered exercise programmes are desirable amongst breast cancer survivors [27] and have demonstrated promise to increase PA amongst cancer survivors [42] and may be a crucial way to reach disadvantaged cancer survivors residing in more rural areas and provide them with tailored support to bridge the gap in access to community-based PA programmes [43]. Despite the dominant thinking related to supervised exercise, participants highlighted the need for tailored PA programmes that align with survivors’ exercise preferences and support needs. Taken together, future service development could focus on promoting the self-management of PA, alongside capitalising on existing opportunities for intervention in practice and offering a diversity of PA interventions to increase reach and align with the exercise preferences and support needs of cancer survivors.

### Study Limitations

Our study recruited participants within Ireland; therefore, findings may not be generalisable. Further limitations include the exclusion of other healthcare professionals involved in cancer care, such as general practitioners and occupational therapists, and the recruitment of oncologists with an interest in exercise oncology that may not reflect the views of other oncologists. There could also be further detail related to the sociodemographic characteristics of participants to better evidence the experience and diversity of the sample. Strengths of the study include the breadth of stakeholders engaged in the discussions and the capture of a diversity of perspectives and experiences related to PA promotion in cancer nationally.

## 5. Conclusions

Our findings underscore the importance of consistent PA messaging (i.e., all relaying the same message to patients including the PA guidelines, that is, to participate in at least 150 min of aerobic moderate-intensity PA per week (or 75 min per week of vigorous-intensity PA) and muscle-strengthening activities on two or more days a week) across HCPs and highlighting the importance of PA to patients and doing so from the point of diagnosis onwards. Findings also emphasised a need for HCP training including PA counselling in clinic and the development of a PA cancer care pathway. The main barrier to PA promotion was limited access to PA programmes. There was also poor referral to existing exercise programmes. The dominant model was facility-based exercise programmes with considerably less focus on home-based PA. Worthwhile intervention efforts include the promotion of lifestyle-based PA, such as walking, alongside capitalising on existing opportunities for intervention in practice, such as the chemotherapy education sessions run by Daffodil nurses and physiotherapy ward visits following surgery, and offering a diversity of PA interventions that aligns with the varying exercise preferences and support needs of cancer survivors. Despite efforts to recruit a diversity of stakeholders, the sample included some PA advocates. More work is needed to gather data among HCPs that have less interest in PA, including those not engaged in PA promotion to garner a fuller understanding of the barriers to PA promotion. Further research is also needed to explore patient understanding of the value of PA during and following a cancer diagnosis, PA preferences, and support needs and to ascertain such information from cancer survivors who are insufficiently physically active and without a history of exercise participation. Future research should aim to fill these gaps.

## Figures and Tables

**Table 2:** Demographic characteristics of participants.

		*N* (%)
Sex		
	Female	27 (67.5%)
	Male	13 (32.5%)
Participant Role		
	Cancer Support Centre Manager	6 (15.0%)
	Physiotherapist	6 (15.0%)
	Oncology Nurse	5 (12.5%)
	Oncologist	3 (7.5%)
	Consumer	5 (12.5%)
	Academic	5 (12.5.%)
	NCCP Representative	4 (10.0%)
	Exercise Specialist	3 (7.5%)
	Other	3 (7.5%)
Geographical Region		
	Dublin	17 (42.5%)
	Galway	4 (10.0%)
	Donegal	3 (7.5%)
	Waterford	4 (10.0%)
	Monaghan	2 (5.0%)
	Cork	2 (5.0.%)
	Wicklow	2 (5.0%)
	Limerick	1 (2.5%)
	Laois	1 (2.5%)
	Kilkenny	1 (2.5%)
	Missing	3 (7.5%)

**Table 3:** Additional quotes related to themes.

Theme	Sub-Themes/Codes	Further Quotes
Embedding PA into the cancer pathway	Singing from the same hymn sheet	“They have to give the same message that exercise is extremely important part of your journey and keep hearing that no matter where they go” (academic, 1) “If a service user comes into your service everyone is telling them the same thing” (breast cancer survivor, 2) “We need all the HCPs within the hospital on board… and the NCCP have a brochure indicating what we should be working towards” (CSC manager, 6)
	PA as an essential part of treatment	“It really needs to be almost prescribed…I want to see you walking…exercising for at least X amount or at least aiming for it” (breast cancer survivor, 1) “If we could give everyone a Godin leisure time questionnaire…to describe the intensity level of activity they do per week and then using that information to inform what level of support people need” (exercise physiologist, 1). “I’m blue in the face talking about the lifestyle measures that are required…they need to be working towards 30 min a day” (medical oncologist, hospital 12).
	Intervention opportunities	“Getting to people early is important because if you wait until they have finished treatment you are missing out on the benefits that accrue during their treatment” (physiotherapist, CSC 6) “I would not estimate the value of your nursing staff…when someone is getting chemotherapy, they’re in the day ward for a number of hours…they’re in the chair talking to the nurses” (academic, 5) “Other patients need help and need assistance and for those patients they need to have, in the same way as it is standard of care for post MI, you go into your Phase II or Phase III your cardiac rehab programme” (physiotherapist, hospital 7) “When someone has their myocardial infarction…the cardiac rehab pathway almost starts straight away” (academic, 4) “The NCCP actually says that all patients should have the opportunity to see psycho-oncology and nobody in Ireland seems to want to take up the psycho-oncology service, patients I mean…the next best thing and maybe even better is exercise” (radiation oncologist, hospital 11)
Education and training		“Walking is one of the preferable forms of exercise, but actually when I get down to the conversation about somebody who [reports] walking every day, when you actually talk to them about it they are not really pushing themselves…They might be walking for an hour a day but not doing the appropriate intensity to get benefit” (ANP, hospital 1) “The understanding of the benefits of exercise for the patients is just…not out there the way it should be…You get into that conversation when….even in our place, well you say go to the gym or…oh I don’t really feel like it, I will do yoga, as if it is comparable, and it is not, you know” (physiotherapist, CSC 6).
Access to appropriate PA interventions	Limited access to exercise specialists	“The amount of people who bounce back to me who couldn’t do their gym registration because once they heard that they had recent cancer treatment, there was a level of hesitancy [in accepting them]” (physiotherapist, hospital 10).
	Ineffective exercise referral and lack of PA services.	“Healthlink is the software they use and if something was built in there that they could refer to…as a means of referral…sending out paperwork about interventions doesn’t work” (academic, 3) “There should be a referral pathway out to the community, a very strong referral pathway out to the community that are running the specialised programme or that have the expertise…not everyone who comes to us needs that detailed medical assistance…they just need guidance” (manager, CSC 6) “Our research that we did a few years ago showed very clearly that patients wanted to be referred in from the hospital. They had confidence in the hospital staff” (ANP, hospital 3).
Tailored and effective programmes		“Each patient has very individualistic needs…one size doesn’t fit everyone at all. Personalisation is important…also need to address what side effect you’re trying to address and what type of exercise intervention you use to do that” (academic, 4). “We could include exercise for everyone but ensure the level of support is applicable to the individual…whether that is somewhere in their local community or whether they’re completely inactive and having side effects from their treatment and need further support” (exercise physiologist, 1) “They don’t want to sit in with a load of cancer survivors because they don’t identify themselves as cancer survivors. They want to put it behind them and never think about it again” (medical oncologist, Hospital 12) “Being able to offer some kind of triage to get the person to the right service at the end of their treatment” (physiotherapist, hospital 10) “I agree with [name removed] about the group-based programmes that it is not something that you can keep up or sustain, like they run for a certain length of time and then finishes…once the programme finishes, they are not going to sign up for it again so there is limited scope with those” (NCCP, 3). “The point of funding the research in [cancer support] centres was to try and get some sort of an efficacy and the idea being that if we can show efficacy for a programme, then the NCCP might be able to coordinate it nationally and roll it out to all the centres, do a train the trainer model…so that people would be able to avail of the same thing in your local Cancer Support Centre regardless of where you live” (NCCP, 1)

## Data Availability

The authors do not have consent to share the original data publicly.

## Supplementary Materials

The following supporting information can be downloaded at: https://www.mdpi.com/article/10.3390/cancers17050850/s1, File S1: Standards for reporting qualitative research; File S2: Overview of codes generated from each focus group and cross-group analysis.
