# Supplementary material for: Promoting Physical Activity to Cancer Survivors in Practice: Challenges and Solutions for Implementation
Source: Cancers (Basel). 2025 Feb 28;17(5):850. doi: 10.3390/cancers17050850 (PMC11898902; doi:10.3390/cancers17050850)
Supplement: Supplementary file 1 [file cancers-17-00850-s001.zip › cancers-3491640-supplementary.pdf]

Supplementary File S1.

Standards for Reporting Qualitative Research (SRQR)*

http://www.equator-network.org/reporting-guidelines/srqr/

Page/line no(s).

Title and abstract

**Title** - Concise description of the nature and topic of the study Identifying the study as qualitative or indicating the approach (e.g., ethnography, grounded theory) or data collection methods (e.g., interview, focus group) is recommended	1
**Abstract** - Summary of key elements of the study using the abstract format of the intended publication; typically includes background, purpose, methods, results, and conclusions	2

Introduction

**Problem formulation** - Description and significance of the problem/phenomenon studied; review of relevant theory and empirical work; problem statement	3-4
**Purpose or research questio**n - Purpose of the study and specific objectives or questions	4

Methods

**Qualitative approach and research paradigm** - Qualitative approach (e.g., ethnography, grounded theory, case study, phenomenology, narrative research) and guiding theory if appropriate; identifying the research paradigm (e.g., postpositivist, constructivist/ interpretivist) is also recommended; rationale**	8
**Researcher characteristics and reflexivity** - Researchers' characteristics that may influence the research, including personal attributes, qualifications/experience, relationship with participants, assumptions, and/or presuppositions; potential or actual interaction between researchers' characteristics and the research questions, approach, methods, results, and/or transferability	7-8
**Context** - Setting/site and salient contextual factors; rationale**	4-5
**Sampling strategy** - How and why research participants, documents, or events were selected; criteria for deciding when no further sampling was necessary (e.g., sampling saturation); rationale**	6
**Ethical issues pertaining to human subjects** - Documentation of approval by an appropriate ethics review board and participant consent, or explanation for lack thereof; other confidentiality and data security issues	6-8
**Data collection methods** - Types of data collected; details of data collection procedures including (as appropriate) start and stop dates of data collection and analysis, iterative process, triangulation of sources/methods, and modification of procedures in response to evolving study findings; rationale**	6-8

**Data collection instruments and technologies** - Description of instruments (e.g., interview guides, questionnaires) and devices (e.g., audio recorders) used for data collection; if/how the instrument(s) changed over the course of the study	6-8
**Units of study** - Number and relevant characteristics of participants, documents, or events included in the study; level of participation (could be reported in results)	6 & 9
**Data processing** - Methods for processing data prior to and during analysis, including transcription, data entry, data management and security, verification of data integrity, data coding, and anonymization/de-identification of excerpts	7
**Data analysis** - Process by which inferences, themes, etc., were identified and developed, including the researchers involved in data analysis; usually references a specific paradigm or approach; rationale**	9
**Techniques to enhance trustworthiness** - Techniques to enhance trustworthiness and credibility of data analysis (e.g., member checking, audit trail, triangulation); rationale**	9

Results/findings

**Synthesis and interpretation** - Main findings (e.g., interpretations, inferences, and themes); might include development of a theory or model, or integration with prior research or theory	9-18 & 19-21
**Links to empirical data** - Evidence (e.g., quotes, field notes, text excerpts, photographs) to substantiate analytic findings	9-18

Discussion

**Integration with prior work, implications, transferability, and contribution(s) to the field -** Short summary of main findings; explanation of how findings and conclusions connect to, support, elaborate on, or challenge conclusions of earlier scholarship; discussion of scope of application/generalizability; identification of unique contribution(s) to scholarship in a discipline or field	19-21
**Limitations** - Trustworthiness and limitations of findings	21

Other

**Conflicts of interest** - Potential sources of influence or perceived influence on study conduct and conclusions; how these were managed	26
**Funding** - Sources of funding and other support; role of funders in data collection, interpretation, and reporting	4

Reference:

O'Brien BC, Harris IB, Beckman TJ, Reed DA, Cook DA. **Standards for reporting qualitative research: a synthesis of recommendations.** *Academic Medicine*, Vol. 89, No. 9 / Sept 2014 DOI: 10.1097/ACM.0000000000000388

Supplementary File S2. Overview of Codes generated from each Focus Group and cross-group analysis

Focus Group	Codes
1 (n= 10)	Automatic referral/unaware of referral/need simple referral process<br>Embed exercise as normal part of cancer pathway<br>Exercise as an essential part of cancer care<br>Tailored messages for exercise at different stages<br>Patients may not be engaging in PA that really counts<br>Some do not want to be identified as cancer patient in exercise environment<br>PA assessment to be routine care (like blood pressure or blood count)<br>Poor uptake and high attrition to exercise programmes (following referral)<br>HCPs need CPD on PA guidelines/how to coach patients for > PA<br>Referrals taken up by those with history of exercise/strong interest in PA<br>Personalised approach to exercise<br>Coaching psychology approach rather than expert driven<br>Need for information (exercise booklet)<br>Exercise advice/prescription needs to come from everybody- all HCPs<br>Buy in from NCCP with result in Oncologist buy in<br>Access free local opportunities<br>Not all able to access structured/supervised programmes offered by ExWell
2 (n= 8)	Provide simple information pack<br>Referrals work well when it is mandatory<br>Not everyone has the budget to attend a gym<br>Access free local PA opportunities, walking groups<br>Exercise as part of standard treatment and essential<br>Promoting PA is not a priority of clinicians<br>Patients overwhelmed with leaflets/information overload<br>Need for Oncologist buy in<br>Assess PA as a screening tool<br>PA promotion should start at point of diagnosis<br>Could be stronger messaging on exercise during chemo education sessions<br>PA needs to be part of cancer care package<br>Clinicians have role in highlighting importance of exercise & role of self-management<br>No one size fits all: need for tailoring and innovations
3 (n= 7)	Time barrier to exercise referral<br>Referrals from hospital for general support but not for exercise<br>No community exercise programmes for cancer<br>PA those counts (cancer support centres offering primarily yoga and Pilates)<br>Lack of exercise specialists trained in exercise oncology<br>Tailoring of exercise for preferences<br>Exercise should be standard of care<br>Clear and direct PA messaging<br>Some clinicians do not view exercise as a priority<br>Role for nurses in frontline to provide PA information<br>Patients unaware of the importance of exercise<br>Cancer diagnosis as a teachable moment to promote PA<br>PA promotion from diagnosis onwards<br>Medical community intoxicated with drugs
4 (n= 7)	Misperceptions about what counts as exercise<br>Role for prescribed exercise

	Little PA information received by patients<br>Need for training of clinicians on the PA guidelines<br>Central role of Oncologist in promoting PA<br>Looking to other healthcare pathways & models and apply to exercise oncology<br>Need buy in from clinicians<br>Role for nurses in the frontline with patients<br>Patients report lack of guidance on exercise as a barrier to engagement<br>No access to exercise specialists<br>Making referral easier for clinicians<br>PA programmes and services are sparse<br>Need for repeated PA messaging across MDT<br>Barriers to attending facility-based programmes (cost, travel, logistics)<br>Making every contact count<br>Get family member involved alongside the patient<br>Get message across that exercise is safe
5 (n= 8)	Confidence as barrier to exercise/need knowledge or guidance<br>Misperceptions on appropriate exercise/what counts<br>Lack of finances to employ exercise specialists/physiotherapist to run classes<br>Need to get oncologists and oncology nurses all on board<br>Exercise prescriptions given in oncology practice<br>NCCP to provide guidance on PA promotion for clinicians<br>PA should be local and free<br>PA tailoring to needs of patient (single, group, online, face-to-face)<br>Some don't like to identify as cancer survivors<br>PA literature to be given to every new patient<br>Opportunity to promote PA by nurse at the education session (chemo education)<br>Teachable moment, opportunity to take some control and do better<br>Consistent PA messaging from diagnosis from everybody<br>Role for automatic referral to programmes<br>Difficulty in recruiting patients to a hospital-based lifestyle programme<br>Paradigm shift needed, so people do not finish treatment when finished 'active' treatment<br>Embedding PA message throughout patient journey<br>PA assessment and triage<br>Funding is a huge issue for patients to attend community exercise programmes<br>Need an approved set of programmes to roll out nationally<br>Unable to drive due to treatment and attend gyms/PA programmes<br>Develop PA model of care like that for psycho-oncology- stepped model of care<br>Many prefer one-to-one, they don't want groups<br>Thinking outside the box, match them to dog shelters<br>Struggle to access appropriate community-based programmes to send patients<br>Apps and wearables to support exercise compliance
Across Groups	Embedding PA into the cancer care pathway<br>Singing from the same hymn sheet<br>PA as an essential part of treatment<br>Intervention Opportunities<br>Education and training<br>Ineffective exercise referral and lack of PA services.<br>Limited access to exercise specialists<br>Tailored and effective programmes
